# Differences in the Production of Extracellular Polymeric Substances (EPS) and Other Metabolites of *Plenodomus* (*Leptosphaeria*) Infecting Winter Oilseed Rape (*Brassica napus* L.)

**DOI:** 10.3390/metabo13060759

**Published:** 2023-06-17

**Authors:** Artur Nowak, Mateusz Kutyła, Joanna Kaczmarek, Jolanta Jaroszuk-Ściseł, Małgorzata Jędryczka

**Affiliations:** 1 Department of Industrial and Environmental Microbiology, Institute of Biological Sciences, Faculty of Biology and Biotechnology, Maria Curie-Skłodowska University, Akademicka 19, 20-033 Lublin, Poland; artur.nowak@mail.umcs.pl (A.N.); mateusz.kutyla@mail.umcs.pl (M.K.); 2 Department of Pathogen Genetics and Plant Resistance, Institute of Plant Genetics, Polish Academy of Sciences, Strzeszyńska 34, 60-479 Poznań, Poland; jkac@igr.poznan.pl

**Keywords:** *Plenodomus* (*Leptosphaeria*), extracellular polymeric substance (EPS), exopolysaccharide, winter oilseed rape, enzyme activity, IAA, β-glucanase, invertase, siderophore

## Abstract

Species of the genus *Plenodomus* (*Leptosphaeria*) are phytopathogens of the Brassicaceae family, which includes oilseed rape. The spores of these fungi spread by airborne transmission, infect plants, and cause crop losses. The secondary metabolism of *P. lingam* and *P. biglobosus* was studied and compared, with the main focus being on the ability to produce Extracellular Polymeric Substances (EPS). In spite of the 1.5–2-fold faster growth rate of *P. biglobosus* on Czapek-Dox and other screening media, the average yield of EPS in this fungus was only 0.29 g/L, compared to that of *P. lingam* (0.43 g/L). In turn, *P. biglobosus* showed a higher capacity to synthesise IAA, i.e., 14 µg/mL, in contrast to <1.5 µg/mL produced by *P. lingam*. On the other hand, the *P. lingam* strains showed higher β-glucanase activity (350–400 mU/mL), compared to 50–100 mU/mL in *P. biglobosus*. Invertase levels were similar in both species (250 mU/mL). The positive correlation between invertase activity and EPS yield contrasted with the absence of a correlation of EPS with β-glucanase. *Plenodomus* neither solubilised phosphate nor used proteins from milk. All strains showed the ability to synthesise siderophores on CAS agar. *P. biglobosus* exhibited the highest efficiency of amylolytic and cellulolytic activity.

## 1. Introduction

The interaction between plants and microorganisms depends on many environmental factors and products of their secondary metabolism. In response to the presence of microorganisms, plants produce numerous compounds that are secreted into the environment. They have a number of functions and can be responsible for defence against biotic and abiotic stresses, as shown in the case of phenols, terpenes, steroids, alkaloids, or flavonoids [1]. Each of these compounds can act as an attractant for microorganisms from the groups of Plant Growth-Promoting Rhizobacteria (PGPR) and Plant Growth-Promoting Fungi (PGPF), or as a protector against phytopathogens [2]. However, plant secondary metabolism is only partially responsible for the plant–microorganism interaction [3]. The range of compounds produced by microorganisms reveals the information necessary to understand the complex plant–microbe interactions [4]. Of particular interest is the study of metabolites synthesized by strains that benefit plants and the environment, such as fungi of the genus *Trichoderma* [5]. The knowledge of phytopathogens that cause plant diseases seems even more important.

In many ways, the metabolism of phytopathogens is often similar to that of beneficial strains and involves the synthesis of compounds with phosphate-solubilising capacity, siderophores, or phytohormones (IAA—Indole-3-acetic acid, GA—gibberellic acid, CKs—cytokinins), as well as cell wall-degrading enzymes (CWDE) and mycotoxins [6]. These compounds are produced during the interaction of phytopathogens with plants, and their metabolism is indicated by physiological and biochemical changes that accompany these interactions [7]. Each of these compounds has a different function during plant infection. Growth regulators, e.g., IAA, GA, or cytokinins, produced in excessive concentrations disrupt the natural phytohormone balance in plants. This leads to the weakening of defence pathways in plant tissues, thus increasing the chance of infection by pathogens [6]. For example, the excessive synthesis of IAA by *Ustilago maydis* increased the susceptibility of maize tissues. In addition, it caused tissue proliferation at the site of infection, resulting in the formation of undesirable nodules [8,9].

One of the most important determinants of the relationships between plants and phytopathogenic fungi is cell-wall degrading enzymes. CWDE take part in the degradation of cellulose, hemicellulose, and cuticle. The high activity of enzymes from this group, i.e., chitinases, cellulases, proteases, glucanases, amylases, and invertases, facilitates the invasion and expansion of phytopathogenic fungi in plant tissues [4,6]. Phytopathogen species from the genera *Fusarium* sp. or *Rhizoctonia* sp. have high CWDE activity, causing the degradation of the bonds between cell wall components and facilitating the penetration of host tissues. The highest levels of these enzymes are noted in the first stages of infection [10,11,12]. *Leptosphaeria* sp. metabolites representing the chemical class of cyclopiane-type diterpene leptosphin and conidiogenone exhibit antibiotic properties and are able to inhibit the growth of *E. coli*, *P. aeruginosa*, and *S. typhimurium* [13].

Fungi produce thousands of secondary metabolites (SMs) with a wide variety of functions and structures, and these are usually low-molecular weight (LMW) compounds [3,14,15]. EPS, on the other hand, are highly diverse polymeric compounds with molecular weight ranging from a few to thousands of kDa, but usually several hundred kDa. This means that EPS include both LMW and high-molecular weight (HMW) compounds [16].

Fungal SMs can be divided into four main chemical classes: polyketides, terpenoids, shikimic acid-derived compounds, and non-ribosomal peptides. Metabolites representing dioxopiperazines, depsipeptides, polyketides, and sesquiterpenes were detected in liquid cultures of various *Leptosphaeria biglobosa* isolates, while sterols and numerous other metabolites not classified in a specific class, such as maculansins and cerebrosides, were detected in *Leptosphaeria maculans* cultures [17].

Mycotoxins are products of secondary metabolism that cause damage to plant tissues and disrupt the entire molecular machinery of infected plants. They affect the processes of protein synthesis and gene expression. Toxins produced by fungi can accumulate at different trophic levels, adversely affecting human health. The most important are aflatoxins, ochratoxins, fumonisins, and deoxynivalenol [18]. *Plenodomus lingam* (*Leptosphaeria maculans*) is a known producer of sirodesmins, which are also regarded as strong fungal toxins with an adverse effect on host plants [19,20]. Metabolites that cause damage to plant tissues (phytotoxins) can be host-selective, i.e., they cause damage to the host plant only, or non-host selective, i.e., harmful to a wide range of plants. The production of phytotoxic metabolites is common in fungi [17]. Metabolites classified as phytotoxins and those not inducing plant damage can act as elicitors, i.e., compounds that induce defence responses in both host and non-host plants [3,21].

The secondary metabolite eutypin secreted by the fungus *Eutypa lata* associated with Grapevine Trunk Disease (GTD) activated defence responses, as evident in extracellular alkalinisation and the induction of phytoalexin synthesis defence genes, such as phenylalanine ammonium lyase (PAL), resveratrol synthase (RS), and stilbene synthase (StSy), and the jasmonate response gene – jasmonate zim domain (JAZ1) [22,23]. In the case of GTD, the EPS turned out to be essential for pathogenicity [3,22,24].

An interesting group of compounds produced by fungi are extracellular polymeric substances (EPS). These are long-chain compounds whose main core is made up of carbohydrate polymers linked to protein, amino acid, lipid, phenolic, or organic acid substituents [16]. The variation in the structure of these compounds, particularly the carbohydrate core, is responsible for their bioactive properties [25]. It can be linked by α- and/or β-type bonds consisting of sugar subunits. In the chain structure, there may be either one type of sugar subunit (homopolysaccharides), or two or more (heteropolysaccharides). The most common sugar monomers in the chain are glucose, galactose, mannose, or rhamnose [26]. They facilitate the adaptation of microorganisms to the environment [27]. These compounds have antioxidant, antibacterial, and antifungal properties [28]. The ability of phytopathogenic strains to synthesise EPS has an impact on the plant infection process. EPS obtained from *Botrytis cinerea* was reported to affect jasmonic acid (JA) and salicylic acid (SA) synthesis pathways in tomato tissues, facilitating the development of grey mould [29]. EPS obtained from axenic cultures of *Fusarium fujikuroi* showed adverse activity against *Cucumis sativus*, causing such symptoms as necrosis, chlorosis, or the yellowing of leaves [30]. The ability to synthesise EPS itself may also depend on strain interactions with plants. For example, a *Fusarium culmorum* strain characterised as a PGPF synthesised EPS at the lowest level of 0.2 g/L, compared to a phytopathogen (1.1 g/L) [31].

The ability of phytopathogens to synthesise multiple secondary metabolites as factors that enhance their ability to infest plants is one of the adaptive features of all species. It is important to know the relationships between different metabolites. Intracellular pathways of EPS synthesis dependent on multiple transcription factors and enzymatic pathways have been described in the literature [28,32,33,34]. There are also reports of EPS synthesis by bacteria involving such enzymes as invertase and glycosyltransferases. Invertase hydrolyses the sucrose bond to release an activated glucose molecule, which can be utilised by glycosyltransferases to elongate the polymer chain [32,35]. However, can such a relationship between EPS synthesis and other compounds produced by fungi be easily determined? This seems especially important in the case of phytopathogenic fungi, e.g., *Alternaria* sp. [36], *Botrytis* sp. [37], *Fusarium* sp. [38], or *Plenodomus* sp. [39]. Representatives of these species are pathogens responsible for high losses in agriculture and horticulture, as they cause a wide range of diseases [40].

The fungal genus *Plenodomus* represents the diverse class Dothideomycetes within the phylum Ascomycota, and belongs to the most numerous order Pleosporales. *Plenodomus* is widely distributed, but found most typically in temperate climate zones. It belongs to the family Leptosphaeriaceae, which includes either saprophobic or necrotrophic genera infecting above-ground parts of plants, stems, and leaves, and easily spreading through the airborne transfer of spores released from asci produced in the ascolocular developmental stage [41,42].

*Plenodomus lingam* (*Leptosphaeria maculans*) and *P. biglobosus* (*L. biglobosa*) are two closely related pathogenic species, sometimes referred to as siblings, which coexist on Brassicaceae host plants [43,44]. The former one is associated with blackleg or stem canker and is regarded as highly damaging in Canada [19], the UK, and many countries of Europe [45,46,47,48]. *Plenodomus biglobosus* has been shown to have a relatively wide host range, causing infection in oilseed rape, *B. oleracea* (cabbage), and *B. rapa* (pak choi), as well as wasabi [49], by multiple genetic subclades of the fungi *Plenodomus lingam* (formerly *Leptosphaeria maculans*) and *P. biglobosus* (*L. biglobosa*). In spring 2021, phoma-like disease symptoms were observed on leaves and stems of *Eutrema japonicum* (wasabi) crops.

The strain-typing of Polish isolates of *Leptosphaeria maculans* (currently termed *P. lingam*) supported the concept of aggressive and non-aggressive strains at the genomic level [50]. The colonisation of cortex tissues by *P. lingam*, compared to superficial growth along the stem epidermis, was attributed to toxic secondary metabolites, with sirodesmin PL and other sirodesmins as the major compounds responsible for the higher aggressiveness of the fungus [45]. *Leptosphaeria biglobosa* was reported to be a producer of benzoic acid, which also had phytotoxic properties [51]. Both species differed in their enzymatic activity [52,53]. The massive deletion in the Internal Transcribed Spacer region and the differences in β-tubulin helped to elaborate several molecular tests based on end-point PCR [54,55], Loop-mediated Amplified Polymorphic DNA [56], and quantitative PCR [55,56,57,58]. The massive deletion in the Internal Transcribed Spacer region and the differences in β-tubulin helped to elaborate several molecular tests based on end-point PCR 263 [54,55], Loop-mediated Amplified Polymorphic DNA [56], and quantitative PCR [57,58,59,60]. The considerable differences between *P. lingam* and *P. biglobosus* result from the variation in their metabolic capacities [39], which inspired us to carry out further comparisons of their biochemical characteristics.

The aim of this study was to compare the secondary metabolism of *Plenodomus lingam* and *P. biglobosus,* with the main focus on EPS production. The findings may lay the groundwork and shed new light on the differences in their colonisation and growth on host plants, in an attempt to explain why the less virulent *P. biglobosus* immunises oilseed rape plants to resist the highly virulent *P. lingam* [61]. To date, extracellular polymeric substances (EPS), the formation of siderophores, and many other characteristics have never been studied in *Plenodomus* (*Leptosphaeria*), and this work is the first to address this issue.

## 2. Materials and Methods

### 2.1. Characterisation of Strains 

The studies were conducted using fungal strains from the collection of the Department of Pathogen Genetics and Plant Resistance, Institute of Plant Genetics, Polish Academy of Sciences. The isolates of *P. lingam* (PLIGR1, PLIGR2, PLIGR3) were compared to each other and to the *P. biglobosus* subclade “brassicae” (PBIGR1, PBIGR2, PBIGR3). The single spore isolates were obtained in 2021 from the stubble (dry stems) of winter oilseed rape (WOSR) collected at three locations:

PBIGR1 and PLIGR1—WOSR field in Krościna Mała (51°22′33″ N 16°56′36″ E). 

Region—Lower Silesia, county—Trzebnica, municipality—Prusice; 

PBIGR2 and PLIGR2—WOSR field in Pobiedziska (52°28′48″ N 17°16′46″ E).

Region—Great Poland, county—Poznań, municipality—Pobiedziska;

PBIGR3 and PLIGR3—WOSR field in Radostowo (53°59′27″ N 18°43′59″ E).

Region—Pomerania, county—Tczew, municipality—Subkowy.

The inoculum was prepared using a standard protocol [62]. Briefly, single spore isolates were obtained from conidiospores originating from pycnidia excised from the stubble after thorough surface disinfection. Then, 5 μL droplets of highly diluted and vigorously vortexed conidiospore suspensions were placed on the Potato dextrose agar (PDA) medium containing 100 mg/L of streptomycin sulphate (Merck, Darmstadt, Germany) and 50 mg/L of ampicillin (Merck, Darmstadt, Germany). Single colonies were inspected under the microscope and transferred onto fresh media with a sterile needle. The isolates were subcultured until they were free from impurities and other microorganisms, which was checked by growing the fungi in 100 mL flasks containing 20 mL of Czapek-Dox liquid medium. Then, the pure culture was transferred onto a PDA medium, and isolates originating from the hyphal tip of a single colony were transferred to V8 agar medium and kept at 20–22 °C for 21 days under alternating 12 h black (near UV) light (L36W/76, OSRAM, Munich, Germany)/12 h darkness to promote sporulation instead of mycelial growth. To obtain the conidiospore suspension, Petri dishes were flooded with 5 mL of sterile distilled water and scraped with a scalpel. The conidial suspension was adjusted to the concentration of 1 × 10^7^ conidiospores/mL of water, and this solution was used for the inoculation of plants.

### 2.2. Pathogenicity Test 

The pathogenicity of the isolates was evaluated with the cotyledon test, based on the standard methodology [63]. The test involved the use of plants of 12 varieties of winter oilseed rape (*Brassica napus* L.), 5 of which belonged to open pollinated varieties and 7 were hybrids F1. The former group had neither the *Rlm7* resistance gene nor the Adult Plant Resistance (APR37) gene. Four of the hybrid varieties harboured *Rlm7* and three possessed APR37, otherwise termed *RlmS* (Table 1).

The seeds of the tested varieties were planted in plastic trays filled with soil substrate (Klasmann-Deilmann TS1, Geeste, Germany) and placed in the controlled environment of a walk-in chamber at 20–22 °C day and night temperature and 12 h light/12 h dark photoperiod (FireSun 150, M. Rochala, Wrocław, Poland). The inoculation was prepared with four selected isolates, two of each species (*P. lingam* and *P. biglobosus*) had contrasting levels of production of EPS. *P. lingam* isolate PLIGR3 produced higher amounts of EPS (0.75 g/L), whereas PLIGR2 produced 0.49 g/L of EPS. In *P. biglobosus*, the highest rate of production of EPS was shown by isolate PBIGR2 (0.57 g/L), and PBIGR3 was a less efficient producer (0.3 g/L).

Droplets of the spore suspension (5 μL, 1 × 10^7^ conidiospores/1 mL) of each isolate were placed on both halves of the cotyledon of each plant previously punctured with a sterile needle. There were 20 replicates (10 plants) per isolate. Trays containing inoculated plants were covered with plastic lids and kept in darkness for 60 h. Afterwards, the lids were removed, and the plants were sprayed with water to maintain high humidity. The temperature and photoperiod were identical to these set prior to the inoculation.

The screening of disease severity was done 14 days after the inoculation, according to the 0–6 IMASCORE scale [63] comprising six infection classes (Figure S1). In both species, 0 meant no difference with the control plants. In *P. lingam*, scores of 1–3 indicated a small to medium necrotic spot and 4–6 were associated with grey-green tissue collapse without the production of pycnidia (4), with scarce pycnidia (5), or with many such fruiting bodies (6). A mean score below 3 was interpreted as a resistant phenotype and A mean score above 3 was regarded a susceptible phenotype. In *P. biglobosus*, the interpretation of isolate virulence was based on the average size of necrotic spots on the cotyledon. In the case of this pathogen, no grey-green tissue collapse was observed, and the evaluation of the symptoms was based on the size of the yellow halo around the wound and the size of necrosis. A score of 6 was given when the discolouration and necrosis covered the whole area of the cotyledon. The mean score of varieties with high and low degrees of cotyledon infection is misleading; therefore, the overall virulence of each isolate is presented as the total score of all cultivars, which helps to reveal the subtle differences between the isolates tested.

### 2.3. Dynamics of the Production of Extracellular Polymeric Substances (EPS) 

Extracellular polymeric substances (EPS) were obtained from cultures on Czapek-Dox medium composed of sucrose 30 g/L (Chempur, Piekary Śląskie, Poland), peptone 7.5 g/L (BTL, Łódź, Poland), NaNO_3_ 3 g/L (POCH, Gliwice, Poland), KH_2_PO_4_ 1 g/L (Chempur, Piekary Śląskie, Poland), KCl 0.5 g/L (POCH, Gliwice, Poland), MgSO_4_·7H_2_O 0.5 g/L (Chempur, Piekary Śląskie, Poland), and FeSO_4_·7H_2_O 0.01 g/L (POCH, Gliwice, Poland), with an initial pH value of 7.0. The cultures were grown at 20 °C with 120 rpm shaking (Innova 4900, Edison, NJ, USA). They were collected between 4 and 17 days of incubation. The mycelium was separated from the culture liquid by filtration on a cellulose filter. EPS was precipitated from the solution using 96% ethanol (Linegal Chemicals, Blizne Łaszczyńskiego, Poland) in a 1:1 ratio for 24 h at 4 °C. After this time, EPS was separated from the supernatant by centrifugation at 10,000 rpm, 15 min and 4 °C (MPW 350-R, Warszawa, Poland), and dried at 75 °C for 8 h in three cycles (Laboratory dryer MOV–112S, Sakata, Oizumi–Machi, Ora–Gun, Gunma Panasonic, Japan). The EPS synthesis yields were expressed in g/L or mg/g [31].

### 2.4. Fungal Strain Growth Dynamics

The growth dynamics of the strains, mycelial dry weight gain, and the final pH value of the culture liquid were determined in the cultures described in Section 2.3. The mycelium obtained was dried at 75 °C for 8 h in three cycles (Laboratory dryer, Philips, Japan) and then weighed (RadWag WPS60K/10, Radom, Poland). The weight was presented in grams of mycelial dry weight per litre of culture (g/L). Before alcohol precipitation, the final pH value was determined in the obtained culture liquid using a pH-meter (Orion 525A, Thermo Electron Corporation, Waltham, MA, USA).

### 2.5. β-Glucanase and Invertase Activity

The β-glucanase activity was determined in supernatants after 3 h incubation with 0.1% solution of laminarin (ratio 1:1) from *Laminaria digitata* (Sigma-Aldrich, St. Louis, MO, USA) in 100 mM acetate buffer (pH 5.6) [64].

The invertase activity was determined in supernatants after 3 h incubation with 0.5% solution of sucrose (ratio 1:1) (Chempur, Piekary Śląskie, Poland) in 100 mM acetate buffer (pH 5.6) [65].

The concentration of reducing sugars was determined using the DNS method [66] with slight modification. Briefly, 1.5 mL of DNS reagent (composed of 0.53% of 3,5-dinitrosalicylic acid (Sigma-Aldrich, St. Louis, MO, USA), 0.99% of NaOH (Sigma-Aldrich, Stockholm, Sweden), 0.415% of NaSO_3_ (Chempur, Piekary Śląskie, Poland), 15.3% of (C_4_H_4_O_6_)KNa·4H_2_O (Stanlab, Lublin, Poland), and 0.38% of phenol (POCH, Gliwice, Poland) in deionised water) was added to 0.5 mL of a properly diluted post-reaction solution. The tubes with the mixture were boiled for 5 min and cooled, and 3 mL of deionised water was added to each tube. The absorbance was measured at λ = 550 nm using an Infinite 200 PRO TECAN microplate spectrophotometer (Tecan, Grödig, Austria), and the number of µmoles was read from the standard curve. One unit of enzyme activity (U) was defined as the amount of enzyme releasing 1 µmol/min of glucose. Extracellular enzyme activity was expressed as mU/mL of post-culture liquid.

### 2.6. Metabolic Activity of Strains—Screening Studies

All screening tests were conducted on solid agar medium at 20 °C. During culture growth, the diameter of fungal colony growth and the diameter of the emerging Echo zone of substrate consumption in the medium were determined [67]. 

The growth rate ratio was determined on all tested media. On each incubation day, the diameters of the colonies were measured, and the R factor of the growth rate was calculated using the formula below and presented as mm^2^ mycelium/day:

$$R=\frac{\left[{\left(\frac{D}{2}\right)}^{2}-{\left(\frac{d}{2}\right)}^{2}\right]\times \pi }{T}$$

where *R*—growth rate factor; D—diameter of the mycelium (mm); d—mycelial discs (11 mm); π—3.14; T—incubation time (day).

The growth factor was calculated on each incubation day, and then the average growth rate ratio over time was calculated (ΔT) and presented as mm^2^/day:

ΔT = ((R_1_ − R_0_) + (R_2_ − R_1_) + … + (R*_n_*_+1_ − R*_n_*))/*n*

where ΔT—growth rate ratio; R—growth rate factor from each day; *n*—number of incubation days.

Effectiveness (E) in screening tests was determined by the difference in the size of the emerging Echo zone and the size of the grown fungal colony:

E = ØEz/ØFc

where E—effectiveness; ØEz—diameter of Echo zone; ØFc—diameter of fungal colony.

Effectiveness was described according to the following scale:

“−” no activity; 

“+” activity level 0.1–1; 

“++” activity level 1.1–2; 

“+++” activity level 2.1–3; 

“++++” activity level 3.1–4;

“+++++” activity level above 4.1.

#### 2.6.1. Proteolytic Activity

The proteolytic activity was determined as the ability of the fungal strains to degrade protein from milk. The culture was carried out on skimmed milk agar (SM agar) containing meat extract 15 g/L (BTL, Łódź, Poland) and skimmed milk 200 mL/L (Łaciate, Mlekpol, Poland) [68].

#### 2.6.2. Cellulolytic Activity

The cellulolytic activity was determined as the ability of the fungal strains to degrade carboxymethylcellulose (CMC). The culture was carried out in CMC agar containing carboxymethylcellulose (CMC) 10 g/L (Sigma-Aldrich, St. Louis, MO, USA), NaNO_3_ 6.5 g/L (POCH, Gliwice, Poland), K_2_HPO_4_ 6.5 g/L (POCH, Gliwice, Poland), yeast extract 0.3 g/L (Difco, Sparks, USA), KCl 6.5 g/L (POCH, Gliwice, Poland), MgSO_4_·7H_2_O 3.0 g/L (Chempur, Piekary Śląskie, Poland), and agar 15 g/L (Biomaxima, Lublin, Poland). After the growth period, 1% Congo Red (Park Scientific, Northampton, UK) was added to the plates and incubated for 30 min at 20 °C. The excess dye was then poured off and the plates were flooded again with 1 M NaCl (POCH, Gliwice, Poland) to wash off the excess dye for 30 min at 20 °C. After this time, the excess solution was poured off and the emerging Echo zones were observed [69].

#### 2.6.3. Phosphate-Solubilising Capacity

The phosphate-solubilising activity was determined as the ability of the fungal strains to degrade insoluble phosphate forms. The culture was conducted on PS medium (PS agar) containing glucose 10 g/L (Chempur, Piekary Śląskie, Poland), asparagine 1 g/L (POCH, Gliwice, Poland), casein hydrolysate 0.2 g/L (POCH, Gliwice, Poland), MgSO_4_·7H_2_O 0.4 g/L (Chempur, Piekary Śląskie, Poland), K_2_SO_4_ 0.2 g/L (POCH, Gliwice, Poland), and agar 15 g/L (Biomaxima, Lublin, Poland) in 800 mL of H_2_O. Solutions of Na_3_PO_4_·12H_2_O 10 g/100 mL (POCH, Gliwice, Poland) and CaCl_2_ 22 g/100 mL (POCH, Gliwice, Poland) were sterilised separately and added after autoclaving (MLS3781L, Panasonic, Japan) [70].

#### 2.6.4. Amylolytic Activity

The amylolytic activity was determined as the ability of the fungal strains to degrade starch. The culture was conducted on Starch medium (AM agar) containing soluble starch 10 g/L (POCH, Gliwice, Poland), KH_2_PO_4_ 0.5 g/L (Chempur, Piekary Śląskie, Poland), K_2_HPO_4_ 0.5 g/L (Chempur, Piekary Śląskie, Poland), MgSO_4_·7H_2_O 0.2 g/L (Chempur, Piekary Śląskie, Poland), (NH_4_)_2_SO_4_ 0.2 g/L (POCH, Gliwice, Poland), and agar 15 g/L (Biomaxima, Lublin, Poland). After the growth period, Lugol’s iodine (POCH, Gliwice, Poland) was added to the plates and incubated for 30 min at 20 °C. After this time, the excess solution was poured off and the emerging Echo zones were observed [71].

#### 2.6.5. Siderophore Synthesis Capacity

The medium used to determine the ability to produce FeCCs was prepared according to the method proposed by Schwyn and Neilands [72]. The CAS agar was prepared in 860 mL of 0.1 M PIPES buffer (Merck, Darmstadt, Germany). The medium contained glucose 4.0 g/L (Chempur, Piekary Śląskie, Poland), KH_2_PO_4_ 3 g/L (Chempur, Piekary Śląskie, Poland), NaCl 0.5 g/L (POCH, Gliwice, Poland), NH_4_Cl 1 g/L (POCH, Gliwice, Poland), MgSO_4_·7H_2_O 0.2 g/L (Chempur, Piekary Śląskie, Poland), and agar 15.0 g/L (Biomaxima, Lublin, Poland). Solutions of 10% acidic casein hydrolysate (POCH, Gliwice, Poland) (30 mL), 0.01 M CaCl_2_ (POCH, Gliwice, Poland) (10 mL), and a dark blue solution of CAS-complex (100 mL) prepared by mixing 60.5 mg chromazurol S (CAS) (Fulka, Göteborg, Sweden) (50 mL), 1 mM FeCl_3_·6H_2_O (POCH, Gliwice, Poland) in 10 mM HCl (POCH, Gliwice, Poland) (10 mL), and 72.9 mg of detergent—hexadecyltrimethylammonium bromide (HDTMA) (Sigma-Aldrich, Hamburg, Germany) (40 mL) were sterilised separately and added after autoclaving (MLS3781L, Sakata, Oizumi–Machi, Ora–Gun, Gunma Panasonic, Japan).

### 2.7. Determination of IAA Concentration

The concentration of IAA was determined using Salkowski’s reagent (1.2% FeCl_3_·6H_2_O) (POCH, Gliwice, Poland) in 7.9 M H_2_SO_4_ (Chempur, Piekary Śląskie, Poland) [73]. The reaction was carried out in a 96-well plate by mixing 100 µL of the supernatant and 100 µL of Salkowski’s reagent. The mixture was intensively mixed for 2 min (MB100-4A, AllSheng, Hangzhou, China). The absorbance of the pink colour that developed after 30 min of incubation in darkness at 20 °C was read at λ = 530 nm using an Infinite 200 PRO TECAN microplate spectrophotometer (Tecan, Grödig, Austria). The IAA concentration was expressed as µg of IAA/mL of post-culture liquid.

### 2.8. Statistical Analysis

The experiments were carried out in triplicate. The data are presented as the mean value with standard deviation (SD). The results were subjected to an analysis of variance (ANOVA) followed by Tukey’s post hoc test for multiple comparisons at *p* < 0.05. Correlations between data obtained after the culture (day, pH, biomass, EPS yield, IAA, β-glucanase, and invertase activity) were analysed based on Pearson’s correlation coefficient *R*. The data were also analysed using PCA (Principal Component Analysis). The statistical analysis was carried out using open-source RStudio for Windows version 2023.03.0 + 386 (Posit, PBC, GNU Affero General Public License v3).

## 3. Results

### 3.1. Dynamics of EPS Synthesis

**Figure 1:**
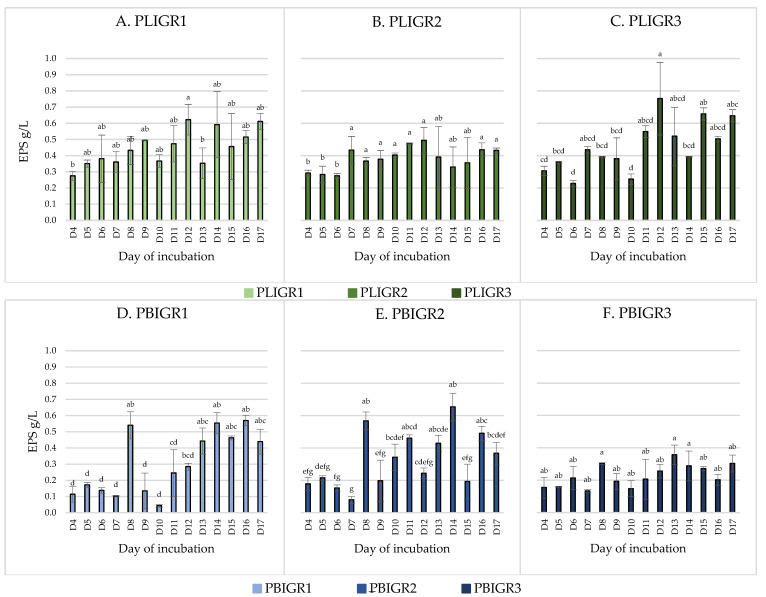
Dynamics of EPS (g/L) synthesis by *Plenodomus lingam* (PLIGR1, PLIGR2, PLIGR3) and *Plenodomus biglobosus* (PBIGR1, PBIGR2, PBIGR3) strains during the culture growth period of 4–17 days. Statistical data analysis: one-way ANOVA with post hoc Tukey’s HSD test, *p* < 0.05. Bars with the different letter are statistically significantly different from each other. Standard deviations are shown as deviation bars (*n* = 3).

The EPSs’ production ability and the dynamics of synthesis over time were tested. EPSs were obtained from cultures of both *Plenodomus lingam* (PLIGR1, PLIGR2, PLIGR3) and *Plenodomus biglobosus* (PBIGR1, PBIGR2, PBIGR3) (Figure 1). The synthesis efficiency differed during culture growth and between the isolates tested.

In the case of the *P. lingam* strains, EPS was obtained throughout the culture growth period (from 4 to 17 days) (Figure 1). The average EPS yield in this species was 0.46 g/L for PLIGR1, 0.38 g/L for PLIGR2, and 0.46 g/L for PLIGR3, respectively (average of all days). Within the entire species, the average EPS synthesis yield was 0.43 g/L (average of all strains). The 12-day incubation period yielded the highest amount of EPS obtained from the culture of this species. EPS production yields of 0.62 g/L for PLIGR1 (Figure 1A), 0.49 g/L for PLIGR2 (Figure 1B), and 0.75 g/L for PLIGR3 (Figure 1C) were obtained in the cultures of the tested strains. The extension of the culture growth period in the tested strains did not cause a statistically significant increase in the amount of EPS obtained from the cultures (Figure 1).

The yield of EPS produced by *P. biglobosus* was approximately 30% lower than that in the *P. lingam* cultures. The highest amount of EPS was obtained after 8 days of culture incubation. The average yield over time was 0.3 g/L for isolate PBIGR1, 0.33 g/L for PBIGR2, and 0.23 g/L for PBIGR3 (average of all days). The yield for the whole species was 0.29 g/L (average of all strains). The highest EPS concentration (day 8 of the culture incubation) was 0.54 g/L—PBIGR1 (Figure 1D), 0.57 g/L—PBIGR2 (Figure 1E), and 0.3 g/L—PBIGR3 (Figure 1F). Subsequently, a decrease in the amount of EPS obtained from the cultures was observed, and again an intensive increase in the EPS concentration was observed in long-term cultures, i.e., between days 13 and 17 of incubation. Similarly to *P. lingam*, the extension of the incubation time of the culture did not result in a statistically significant increase in the EPS concentration obtained from the culture (Figure 1).

**Figure 2:**
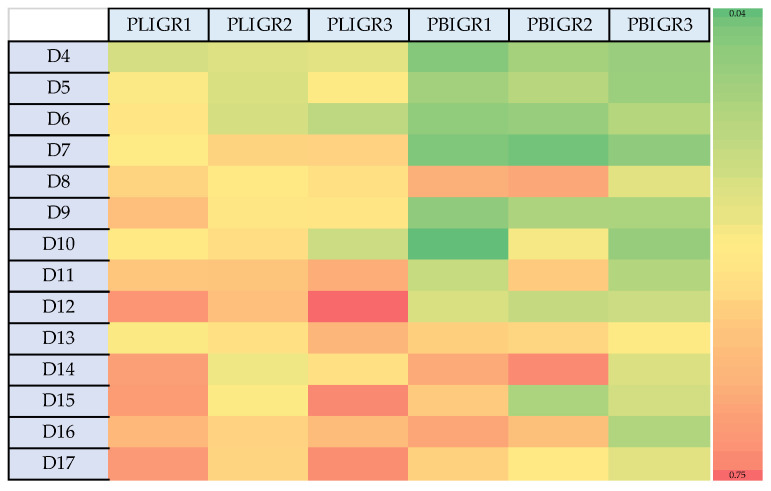
Heat map presenting the differences in the EPS synthesis dynamics between *Plenodomus lingam* (PLIGR1, PLIGR2, PLIGR3) and *Plenodomus biglobosus* (PBIGR1, PBIGR2, PBIGR3) species during the 4–14-day culture growth. The colour intensity on the heat map corresponds to the EPS g/L synthesis efficiency.

The heat map (Figure 2) shows the variation in EPS production by the fungal strains. *Plenodomus lingam* produced significantly greater amounts of EPS than *P. biglobosus*. In addition, an increase in the EPS production was exhibited by the *P. lingam* strains on day 12 of culture growth. In the case of the *P. biglobosus* strains, the greatest increase in EPS synthesis intensity was noted on day 8, and then another increase in EPS levels was observed from day 14 onwards.

### 3.2. Fungal Strain Growth Dynamics

**Figure 3:**
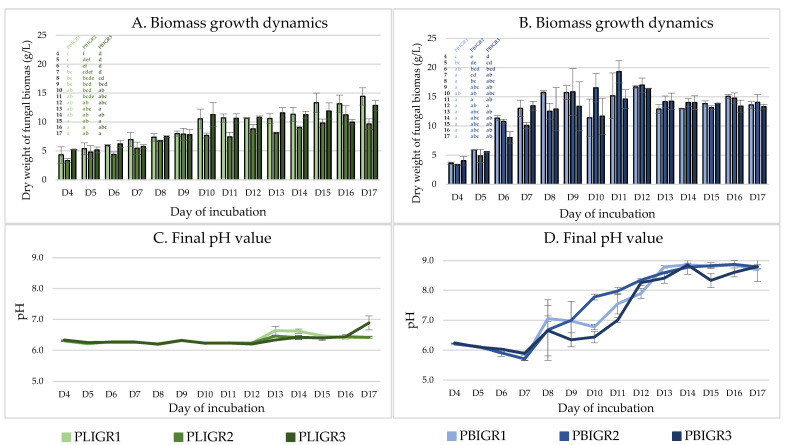
Dynamics of fungal biomass growth and changes in the pH value in *Plenodomus lingam* (PLIGR1, PLIGR2, PLIGR3) and *Plenodomus biglobosus* (PBIGR1, PBIGR2, PBIGR3) strains during culture growth for 4–17 days. Statistical data analysis: one-way ANOVA with post hoc Tukey’s HSD test, *p* < 0.05. Bars with the different letter are statistically significantly different from each other. Bars with the different letter are statistically significantly different from each other. Standard deviations are shown as deviation bars (*n* = 3).

The growth dynamics in the tested strains were determined by assessment of the increase in biomass during culture growth and changes in the final pH value of the culture liquids (Figure 3).

The increase in the biomass of *P. lingam* strains was consistent throughout the culture incubation period, reaching a maximum between incubation days 15 and 17 (Figure 3A). The slowest biomass growth was exhibited by the PLIGR2 strain, where 9.6 g/L of biomass was obtained. For strains PLIGR1 and PLIGR2, the highest biomass growth (12.8–14.4 g/L) was observed on day 17. A similar relationship was observed for the final pH value, where the pH value during the first 11 days of incubation ranged from 6.2 to 6.7. An increase in the final pH value above this level was observed only on day 13 and the following days. Finally, the final pH value remained at the level of slight acidification of the medium, which is characteristic of the stage of intensive growth of filamentous fungi (Figure 3C).

The biomass growth dynamics in the *P. biglobosus* strains were significantly higher than those of *P. lingam*. The maximum biomass growth of the tested strains was achieved between days 9 and 10 of culture incubation, reaching an average level of 15.8–19.2 g/L (Figure 3B). These values were 1.5 times higher than in the *P. lingam* culture. From day 12, a slow but statistically insignificant decrease in the biomass was observed. This was correlated with an increase in the final pH value of the medium to 7.5–9.0. Such a high increase in the final pH value, together with the decrease in the overall biomass obtained, may indicate that the mycelium is entering the autolysis stage (Figure 3D).

**Figure 4:**
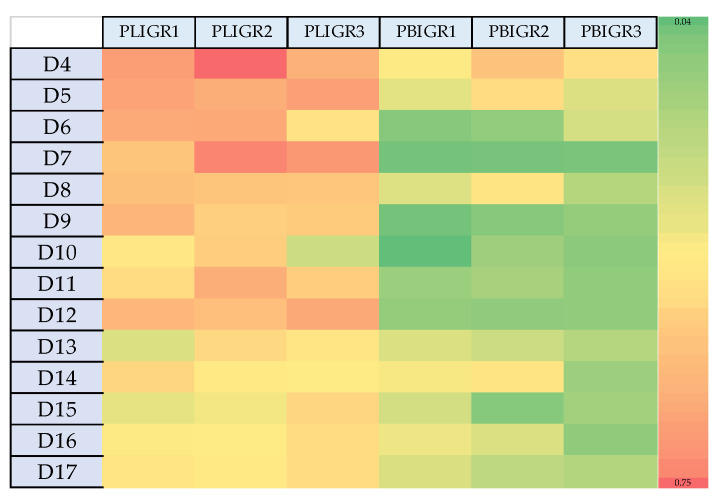
Heat map presenting the differences in the EPS yield and the biomass growth rate (mg/g) between *Plenodomus lingam* (PLIGR1, PLIGR2, PLIGR3) and *Plenodomus biglobosus* (PBIGR1, PBIGR2, PBIGR3) species during the 4–14-day culture growth. The colour intensity on the heat map corresponds to the EPS mg/g synthesis efficiency.

The dependence between the EPS synthesis yield per L of post-culture liquid (g/L) (Figure 2) did not coincide with the EPS yield in relation to the biomass obtained (mg/g). The highest EPS synthesis efficiency in relation to the biomass gain was observed in the early days of culture of the tested strains (Figure 4). This was particularly clear in the case of *P. lingam*. The variation in this g/L to mg/g dependence may be important for large-scale cultures, where the resulting mycelium may represent post-production waste requiring management.

### 3.3. Pathogenicity Tests

The score of the mean infection of the open pollinated cultivars with *P. lingam* was 4.25, with a slight difference between the isolates (PLIGR2 and PLIGR3: 4.00 and 4.05, respectively). The mean disease score for the hybrids possessing *Rlm7* was 1.39, with 1.23 as the mean score obtained for PLIGR2 and 1.55 for PLIGR3. Cultivars with APR37 showed smaller phoma leaf spots. In general, the *P. lingam* PLIGR2 isolate, which produced smaller amounts of EPS, was also slightly less virulent on average (total score 33.83 versus 35.84 for PLIGR3), as shown by the comparison of Figures S2 and S3 in the Supplementary data. The symptoms caused by PLIGR2 were 94.4% less intense than those caused by PLIGR3, but lower virulence was not the case for every cultivar tested (Table 2).

Resistance genes *Rlm7* had no apparent effect on *P. biglobosus*, and the disease symptoms were observed on both open pollinated and hybrid cultivars. In contrast to *P. lingam*, the leaf spots caused by *P. biglobosus* were smaller on the cotyledons of the open pollinated cultivars (mean score 2.20) than on the hybrids (3.60). The mean infection of the hybrids with APR37 was 3.87, which was higher than in the hybrids without APR37 (3.32). *Plenodomus biglobosus* isolate PBIGR 2, which is a more efficient producer of EPS, was on average slightly more virulent (summary score 38.51) than PBIGR3 (33.25). This rule is clear when average results are compared, but it is non-existent or even reversed for the highest scores (Figures S2 and S3). The virulence of isolate PBIGR3 (the least efficient EPS producer) accounted for 86.3% of that of PBIGR2 (Table 2).

### 3.4. Enzymatic Activity

#### 3.4.1. β-Glucanase Activity

The β-glucanase activity was determined in the post-culture liquids of the isolates studied. The representatives of both species showed the ability to synthesise this enzyme. The level of the activity varied between the species.

**Figure 5:**
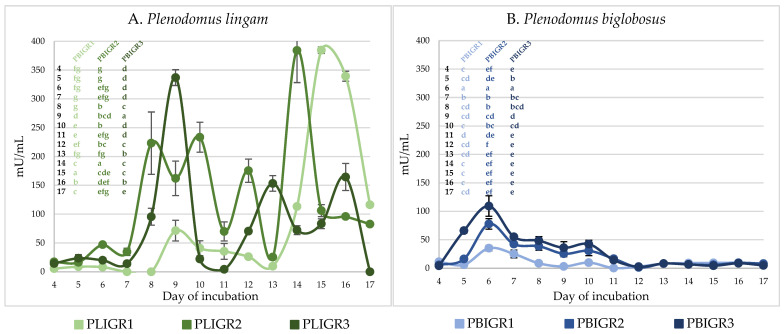
β-Glucanase activity in cultures of (**A**) *Plenodomus lingam* (PLIGR1, PLIGR2, PLIGR3) and (**B**) *Plenodomus biglobosus* (PBIGR1, PBIGR2, PBIGR3) strains. Statistical data analysis: one-way ANOVA with post hoc Tukey’s HSD test, *p* < 0.05. Bars with the different letter are statistically significantly different from each other. Standard deviations are shown as deviation bars (*n* = 3).

The β-glucanase activity of *P. lingam* strains was 2–3 times higher than that of *P. biglobosus* (Figure 5). However, a common trend in the level of this enzyme activity was observed in the *P. biglobosus* strains during the culture period. The isolates of this species showed the highest activity after 6 days of the culture incubation, with levels of 35 mU/mL for PBIGR1, 77 mU/mL for PBIGR2, and 109 mU/mL for PBIGR3. Then, the activity dropped to <10 mU/mL in the long-term cultures (Figure 5B). In turn, a high variation of the activity of this enzyme was demonstrated in the *P. lingam* strains. For PLIGR1, the activity of 384 mU/mL was observed in long-term cultures after 15–16 days of incubation. The activity in the PLIGR2 isolate was 384 mU/mL on day 14 of culture incubation. The highest activity of this enzyme (337 mU/mL) was observed on day 9 of growth only in the PLIGR3 culture (Figure 5A).

#### 3.4.2. Invertase Activity

**Figure 6:**
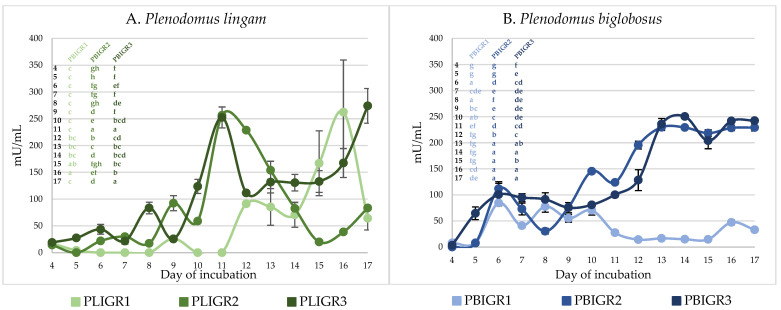
Invertase activity in cultures of (**A**) *Plenodomus lingam* (PLIGR1, PLIGR2, PLIGR3) and (**B**) *Plenodomus biglobosus* (PBIGR1, PBIGR2, PBIGR3) strains. Statistical data analysis: one-way ANOVA with post hoc Tukey’s HSD test, *p* < 0.05. Bars with the different letter are statistically significantly different from each other. Standard deviations are shown as deviation bars (*n* = 3).

The invertase activity observed in the culture liquids of the strains tested also varied both between the species and within the isolates tested. At the peak of activity, the levels of this enzyme were similar (250 mU/mL) in both species studied (Figure 6).

The activity of invertase during the first 7 days of culture incubation was low, i.e., 50 mU/mL, and even <1 mU/mL in the case of the PLGIR1 strain (Figure 6A). From 8 days of culture growth, a definite increase in the activity of this enzyme was observed. In the case of the PLGIR2 and PLIGR3 strains, the peak of activity (250 mU/mL) occurred on culture incubation day 9, but then the activity began to decrease. For strain PLIGR1, a sharp increase in invertase activity was noted on day 11, reaching a maximum level of 262 mU/mL on day 16. For *P. biglobosus*, an increase in invertase activity was observed from culture incubation day 5 to an average level of 50–100 mU/mL in all the isolates tested. This activity persisted until day 10. The invertase activity for PBIGR2 and PBIGR3 increased rapidly to 250 mU/mL and persisted at this level until the end of the culture period. On the other hand, in the case of strain PBIGR1, a rapid decrease in the activity of this enzyme to a level of 15 mU/mL was observed, which was maintained throughout the culture period (Figure 6B).

### 3.5. IAA Concentration

**Figure 7:**
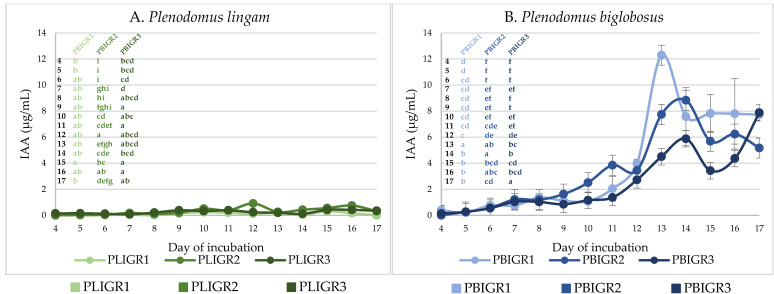
Ability to synthesise the IAA phytohormone by (**A**) *Plenodomus lingam* (PLIGR1, PLIGR2, PLIGR3) and (**B**) *Plenodomus biglobosus* (PBIGR1, PBIGR2, PBIGR3) strains. Bars with the different letter are statistically significantly different from each other. Statistical data analysis: one-way ANOVA with post hoc Tukey’s HSD test, *p* < 0.05. Standard deviations are shown as deviation bars (*n* = 3).

In the case of the *P. lingam* strains, the maximum IAA concentration of 1 µg/mL was observed for the PLIGR2 isolate. The amount of IAA produced by the PLIGR1 and PLIGR2 strains did not exceed 0.8 µg/mL (Figure 7A). However, the isolates of the *P. biglobosus* species showed a significantly higher ability to synthesise IAA, i.e., it was almost 10-fold higher than that of the *P. lingam* strains. The greatest increase in the production of this phytohormone was observed from culture incubation day 12. For strain PBIGR1, the highest concentration of IAA (12 µg/mL) was observed on day 13. In the cases of PBIGR2 and PBIGR3, the highest concentrations of IAA were 8.8 µg/mL and 7.8 µg/mL after 14 and 17 days of incubation, respectively (Figure 7B).

### 3.6. Correlations between EPS Synthesis and Enzymatic Activity

**Figure 8:**
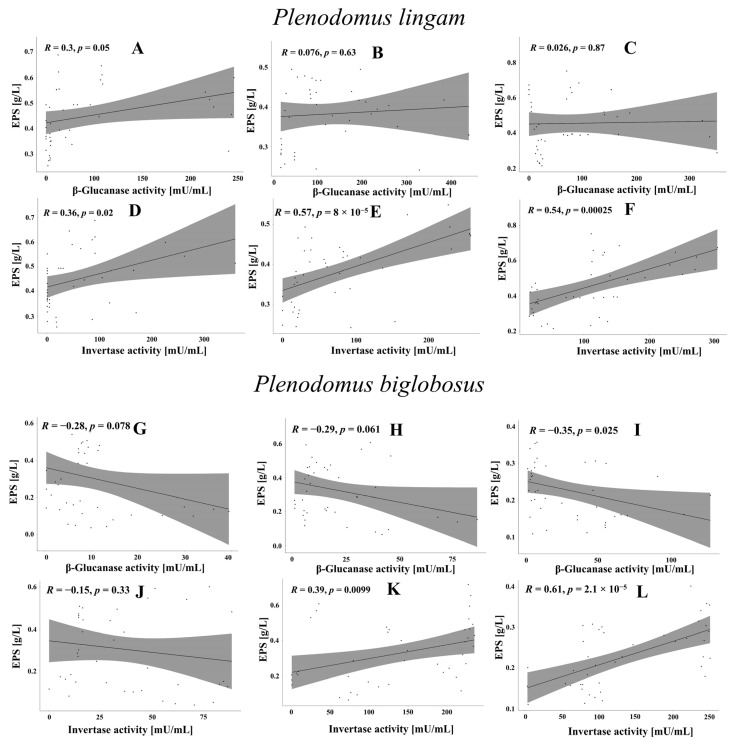
Correlation between EPS synthesis efficiency (g/L) and β-glucanase and invertase activity in PLIGR1 (**A**,**D**), PLIGR2 (**B**,**E**), and PLIGR3 (**C**,**F**), and in PBIGR1 (**G**,**J**), PBIGR2 (**H**,**K**) and PBIGR3 (**I**,**L**). The correlations were analysed based on Pearson’s correlation coefficient *R* and the statistical significance *p* of this coefficient.

In order to determine the effect of β-glucanase and invertase on EPS synthesis, the EPS yields were correlated with the enzyme activity (Figure 8). In the case of the *P. lingam* strains, a statistically significant positive correlation was found between the invertase activity and the EPS yield. There was a positive correlation between the invertase activity and the amount of EPS. The highest value was noted for PLIGR2 *R* = 0.57 at *p* = 8 × 10^−5^ (Figure 8E) and PLIGR3 *R* = 0.54 at *p* = 0.00025 (Figure 8F). A low positive correlation coefficient was noted for PLIGR3, which was *R* = 0.36 at *p* = 0.02 (Figure 8D). The β-glucanase activity was not related to the efficiency of EPS synthesis by the *P. lingam* strains (Figure 8A–C). There was a positive correlation between the invertase activity and the amount of EPS. The highest value was noted for PLIGR2 *R* = 0.57 at *p* = 8 × 10^−5^ (Figure 8E) and PLIGR3 *R* = 0.54 at *p* = 0.00025 (Figure 8F). A low positive correlation coefficient was noted for PLIGR3, which was *R* = 0.36 at *p* = 0.02 (Figure 8D). The β-glucanase activity was not related to the efficiency of EPS synthesis by the *P. lingam* strains (Figure 8A–C). The extracellular invertase activity also influenced the EPS synthesis in the *P. biglobosus* strains. A positive correlation between the invertase activity and the amount of EPS was demonstrated for PBIGR2 (*R* = 0.39, *p* = 0.0099) (Figure 8K) and PBIGR3 (*R* = 0.61, *p* = 2.1 × 10^−5^) (Figure 8L). There was no correlation between the invertase activity and the EPS yield in the PBIGR1 strain (Figure 8J). The efficiency of EPS synthesis by strains PBIGR1 and PBIGR2 was not dependent on the β-glucanase activity (Figure 8G,H). Only the PBIGR2 strain showed a negative correlation between the two variables (Figure 8I).

**Figure 9:**
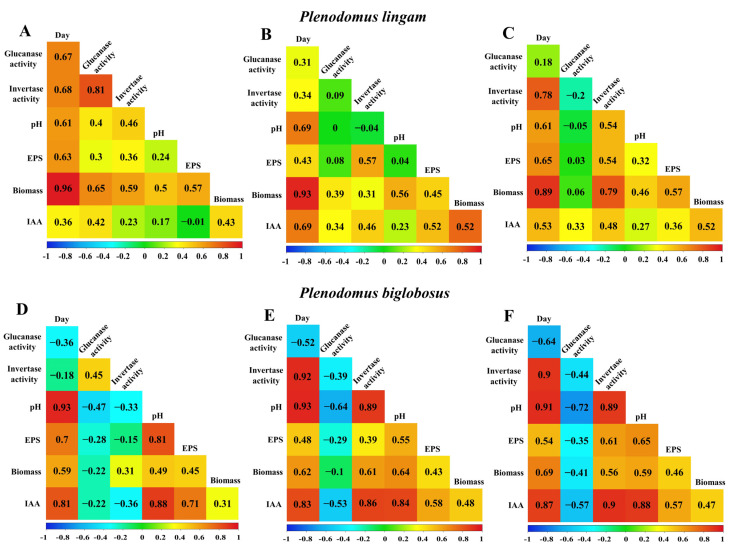
Correlation matrices between day, pH, biomass, EPS yield, β-glucanase, and invertase activity obtained in cultures of PLIGR1 (**A**), PLIGR2 (**B**) and PLIGR3 (**C**), and PBIGR1 (**D**), PBIGR2 (**E**) and PBIGR3 (**F**). The results are presented as Pearson’s correlation coefficient *R*.

In order to fully illustrate the dependence between all variables, correlation matrices were created for each *Plenodomus* strain (Figure 9). In all strains, the amount of biomass obtained increased with the extension of the cultivation time, which had an impact on the other variables through primary and secondary metabolism. In the case of the PLIGR1 strain, the amount of EPS obtained depended mainly on the amount of fungal biomass (*R* = 0.57) and, to a lesser extent, on the invertase activity (*R* = 0.36).

There was a high positive correlation between the invertase and β-glucanase activities (*R* = 0.81). The amount of IAA obtained was positively correlated with the β-glucanase activity (*R* = 0.42) and the amount of biomass (*R* = 0.43) (Figure 9A). In the PLIGR2 and PLIGR1 strains, the yield of EPS synthesis influenced the amount of fungal biomass (*R* = 0.57) and invertase activity (*R* = 0.54). In turn, there was no correlation between the invertase and β-glucanase activity (*R* = −0.2). The amount of IAA obtained was positively correlated with the amount of biomass (*R* = 0.52) and invertase activity (*R* = 0.48). The β-glucanase activity in PLIGR2 was not correlated with any variable (Figure 9B). Similar correlations as in PLIGR2 were also found in the PLIGR3 strain. The EPS synthesis was positively influenced by the invertase activity (*R* = 0.57) and by the amount of biomass (*R* = 0.45), without any influence of the β-glucanase activity (*R* = 0.08). In the PLIGR3 strain, the amount of fungal biomass had the greatest influence on IAA synthesis (*R* = 0.76) (Figure 9C).

In the case of the PBIGR1 strain, the EPS synthesis was dependent on the amount of fungal biomass (*R* = 0.45), without any influence of β-glucanase (*R* = −0.28) or invertase (*R* = 0.31) activity. However, a correlation between the β-glucanase and invertase activities was noted (*R* = 0.45). The increase in the amount of fungal biomass was not reflected in higher levels of IAA synthesis (*R* = 0.31) (Figure 9D). A negative correlation between the β-glucanase and invertase activities was observed in the PBIGR2 strain (*R* = −0.44). The efficiency of the EPS synthesis was positively correlated with the invertase activity (*R* = 0.61) and fungal biomass (*R* = 0.46), and negatively correlated with the β-glucanase activity (*R* = −0.41). The amount of IAA was strongly correlated with the invertase activity (*R* = 0.9) and biomass concentration (*R* = 0.47). The β-glucanase activity was negatively correlated with the variable studied (Figure 9E). Similar correlations to those exhibited by PBIGR2 were also obtained for PBIGR3 (Figure 9F).

**Figure 10:**
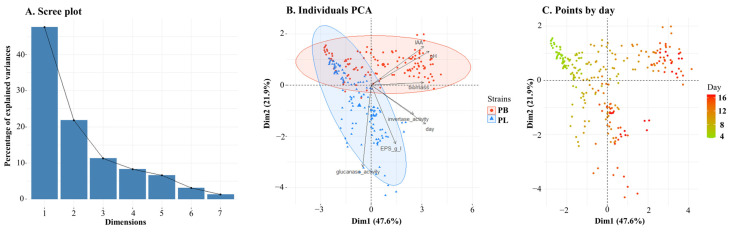
Scree plot (**A**), biplot (**B**) and plot of points by day (**C**) of the Principal Component Analysis (PCA) describing the pH, amount of biomass, day of the culture, IAA and EPS yields, and β-glucanase and invertase activities in *Plenodomus* species.

A PCA analysis was performed to highlight the diversity between the two fungus species. The two species were used as classes, and the values of the day of culture, pH, β-glucanase activity, invertase activity, EPS, biomass, and IAA yield were used as variables. In the first stage, Bartlett’s sphericity test was performed to determine the usefulness of the factor analysis. The working hypothesis that the variables are uncorrelated was rejected at the significance level of 0.001, which justifies the use of the PCA analysis. The cumulative percentage of the explained variance of the analysed variables was used as a selection criterion to reduce the number of principal components. Components Dim1 and Dim2 explained the largest percentage of variance—69.5% in total, with principal component 1 (Dim1) and principal component 2 (Dim2) accounting for 47.6% and 21.9%, respectively (Figure 10A).

The PCA analysis shows that two *Plenodomus* species differ from each other (Figure 10B). The difference between the species is visible during culture. Figure 10C shows the distribution of culture days on the PCA plot. The intersection zone, which proves the lack of differences between the species, occurs at variable values from the initial days of cultivation (D4–D6). After the 7th day of culture, the differentiation of both *Plenodomus* species becomes visible. In the case of *Plenodomus lingam*, the production of EPS is a factor that affects the diversity in this group. Biomass, pH and IAA are the most important variable contributors to Dim1, and they have higher values in *P. biglobosus* isolates, as can also be seen in Figure 3 and Figure 7. β-Glucanase activity and EPS production are the most important variable contributors to Dim2, and they have higher values in *P. lingam* isolates, as can also be seen in Figure 1 and Figure 5. The PCA analysis performed on data from all strains indicates that IAA production is strongly correlated with pH, and invertase activity is correlated with the day of culture (Figure 10B). The close proximity of the variables in the diagram proves their high correlation.

### 3.7. Ability of the Tested Strains to Synthesise Diverse Secondary Metabolites Facilitating Environmental Adaptation

The ability to grow on diverse screening media and the ability to produce metabolites that facilitate adaptation to diverse environmental conditions were determined in all the *Plenodomus* strains (Table 3).

On CAS agar, AM agar, and CMC agar, the average growth rates of the *P. lingam* and *P. biglobosus* strains were lower than <1 mm^2^/day. The *P. lingam* strains showed the lowest growth rate ratio (0.035 mm^2^/day) on CAS agar, whereas the average growth rate for the tested *P. biglobosus* strains was ~0.555 mm^2^/day on this medium (Table 3A). On AM agar and CMC agar, the average growth rate ratio in all the tested strains was at a similar level of 0.4–0.5 mm^2^/day (Table 3B,C). On PS agar and SM agar, the average growth rate ratio of the tested strains was 10-fold higher (Table 3D,E). The highest growth rate ratio was observed in SM agar, where the *P. lingam* strains had an average growth rate of 6 mm^2^/day. Only the PBIGR2 isolate of *P. biglobosus* had an average growth rate ratio of less than 10–12 mm^2^/day, i.e., 5.128 mm^2^/day. On PS agar, the highest growth rate ratio was shown by *P. lingam* strain PLIGR1 (3.337 mm^2^/day) and *P. biglobosus* strain PBIGR1 (7.305 mm^2^/day).

It seems interesting that there was no correlation between the average growth rate ratio and the ability to produce specific secondary metabolites on the tested substrates. The fastest growth rate of the tested strains was observed on PS agar and SM agar, but they did not show the ability to solubilise phosphate (−) and to use the protein from the milk in SM agar (−) (Table 4D,E).

All the strains showed the ability to synthesise siderophores on CAS agar. However, the *P. biglobosus* strains showed 2-fold higher efficiency in producing siderophores (+++++) (Table 4A). In the case of amylolytic (+++) and cellulolytic (++++) activities, the highest efficiency was shown by strain PBIGR1 (Table 4B,C). The *P. lingam* strains showed similar efficiency in producing secondary metabolites on the tested media (++) (Table 4A–C).

## 4. Discussion

The study revealed some important unknown information about the metabolism of *P. lingam* and *P. biglobosus*, including the production of Extracellular Polymeric Substances (EPS). We have demonstrated that the six tested strains belonging to the species *P. lingam* and *P. biglobosus* have the ability to secrete EPS into the medium. The three *P. lingam* strains synthesised significantly greater amounts of EPS than *P. biglobosus*. The mean EPS concentration from 12 days of culture was 0.43 g/L for *P. lingam* and 0.29 for *P. biglobosus*. Thus, the EPS concentration of the *P. biglobosus* culture corresponded to 66.7% of the EPS concentration in *P. lingam*. The mean EPS concentration for the whole genus *Plenodomus* was 0.36 g/L. The ranges of the highest EPS yields obtained in the cultivation of Asco- or Basidiomycota fungi are variable, but are usually approximately 0.1–1.0 g/L. Within the Ascomycota, there are very few strains in which the EPS concentration exceeds 10 g/L. These include the strain *Aureobasidium pullulans* RYLF-10 producing up to 40 g of EPS in 1 L of culture [16,74]. The efficiency of EPS production is a strain feature depending on the age of the culture, temperature, and substrate composition. 

The EPS concentrations obtained in the cultures of *Plenodomus* were similar to these found in cultures of numerous non-pathogenic and phytopathogenic species of Ascomycota, e.g., *Aspergillus* (0.2–0.6 g/L)—*Aspergillus versicolor* (0.24 g/L), *Penicillium commune* (0.43 g/L), *Botryosphaeria rhodina* (0.4 g/L), *Fusarium oxysporum* (0.21–0.59 g/L) [16], and *F. culmorum* (approx. 0.2–1.0 g/L) [31]. The EPS concentration (0.2 g/L) obtained in cultures of a non-pathogenic PGPF *F. culmorum* strain was three times lower than in the DRMO culture and five times lower than in the culture of the pathogenic *F. culmorum* fungus [20]. 

Strain CCFEE 5080 of the Ascomycota filamentous fungus *Phoma herbarum* (Pleosporales order) isolated from continental Antarctica soil produced EPS on a variety of carbon sources in optimal conditions (sorbitol as a C source, NaNO_3_ as a N source, and 28 °C temperature of incubation), with the yield reaching a level of 13.6 g/L [75].

Endophytic Ascomycota fungi isolated from *Piper hispidum*: *Phoma herbarum* and three *Diaporthe* sp. (Diaporthales order) strains secreted EPS at 72, 96, and 168 h of incubation, respectively, and the EPS yield at the 96 h incubation, which was optimal for EPS production, was 2.7 g/L in *P. herbarum* culture and 7.9, 17.6, and 10.9 g/L, respectively, in the cultures of the *Diaporthe* sp. strains [76].

*Plenodomus lingam* and *P. biglobosus* differ in the mode and strength of infection and the location of disease symptoms on a plant. Our study has shown that, although they are called siblings [77] and referred to as a “species complex” [78], both species clearly differ in their metabolic potential. The *Plenodomus biglobosus* strains produced higher biomass, and their pH values of the post-culture fluid were also higher than in the *P. lingam* cultures. Additionally, the growth rate of *P. biglobosus* on siderophore and phosphate dissolution screening media was significantly higher than that of *P. lingam*. Such cyclic changes in biomass and pH values were observed in cultures of other Ascomycota strains. Particularly high pH values were found in cultures of *F. culmorum*, but only on substrates with a plant cell wall as a carbon source [11].

*Plenodomus biglobosus* showed significantly higher cellulolytic activity, and some strains had the ability to synthesise both siderophores and amylolytic enzymes. Amylolytic and cellulolytic activity facilitating the colonisation of plant tissues is typical for endophytic fungi, regardless of their effect on plants, including growth-promoting strains [79]. The activity of these enzymes was detected in some species isolated from maize, i.e., *Cladosporium*, *Aspergillus*, and *Penicillium*, but was absent in different species of the genus *Fusarium* [80].

Siderophores produced by *Plenodomus* strains belong to one of two major hydroxamate-based peptidyl siderophores employed by fungi: depsipeptides and the coprogen family of siderophores. The ability to produce siderophores is a characteristic trait of pathogenic fungal species, e.g., the rice blast fungus *Magnaporthe oryzae*, responsible for their virulence [81,82].

The species *P. biglobosus* had a much greater ability to produce the phytohormone auxin IAA than *P. lingam*. Auxins produced by *Plenodomus* strains not only affect plant growth, but also regulate the defence response of plants [83]. The concentrations of auxin IAA and gibberellins were significantly higher in cultures of *F. culmorum* (PGPF) that were non-pathogenic to cereals, compared to harmful or pathogenic strains [84]. On the other hand, *P. lingam* had a much higher activity of β-glucanase, although this activity changed strongly and cyclically during culture. The species *P. biglobosus* cultured for over 12 days nearly lost its β-glucanase activity.

Based on the fungal ability to utilise substrates in Biolog FF plates, it was demonstrated [39] that the less specialised *P. biglobosus* species used a significantly greater number (34–48) of substrates than *P. lingam*, using 25–29 carbon sources. The finding allows speculations that *P. lingam* coevolves more strictly with the host plant, which coincides with suggestions that this species is evolutionarily younger [85,86]. Indeed, *P. lingam* is better adapted to oilseed rape and, hence, also more aggressive towards plants.

Before day 6 there was no major variation between the fungal biomass, the pH of the culture filtrate and the levels of β-glucanase, invertase and EPS production by *P. lingam* and *P. biglobosus*. However, these characters dramatically change in time, and there was a significant difference between the two species, increasing with each day of the culture in axenic conditions, which makes them easy to distinguish at the biochemical level.

It is very likely that the metabolites synthesised by *Plenodomus* isolates can influence plant growth and health, either on their own or through interactions with other metabolites. Such a synergistic effect was observed for two 3,4-dihydroisocoumarins—(3R,4R)-4-hydroxymellein and (3R,4S)-4-hydroxymellein —produced by *Sphaeropsis sapinea* pathogenic to *Pinus radiata* [87].

The pathogenicity test with *P. lingam* revealed the effectiveness of the *Rlm7* resistance gene and a lower degree of protection of winter oilseed rape plants by APR37 (*RlmS*). All the open pollinated cultivars with no *Rlm7* were severely infected by *P. lingam*, compared to the hybrids with the *Rlm7* resistance gene. This finding is in line with previous studies showing the effectiveness of oilseed rape protection with *Rlm7*; a Europe-wide study of *L. maculans* (*P. lingam*) isolates collected in 2002 detected virulent *avrLm7* isolates at only one site in Sweden [62]. No effect of these resistance genes on *P. biglobosus* was found. The inoculation of plant cotyledons with *P. biglobosus* caused disease symptoms in both the open pollinated and hybrid cultivars. Strangely, the phoma leaf spots on the hybrids with APR37 were slightly bigger in comparison to the symptoms on the hybrids with no APR37. Apparently, Adult Plant Resistance has no effect at the cotyledon stage. It is noteworthy that the better producers of EPS in both *P. lingam* and *P. biglobosus* were also slightly more virulent towards winter oilseed rape plants. This general rule had several exceptions, as shown in this study, but more research is needed to confirm the trend. The extracellular production of EPS in axenic cultures does not ensure their production on or in the tissues of the host plant. However, these compounds are surely produced for a reason; hence, the engagement of EPS in the infection process is a possible explanation.

As part of the matrix of biofilms formed by both single and multiple species and genera, microbial exopolymers can be included among the main determinants of the colonisation of the surface of plant organs, which is the first stage of every infection [16,31]. On the other hand, EPS treated with lytic enzymes, such as glucanases, are the source of short fragments composed of several monomers that can act as elicitors of plant resistance [31,64]. The effectiveness of elicitors depends on their structure and may be limited, for example, by α-glucans [88,89]. It can be assumed that the EPS components produced by *Planodomus* sp. may be effective elicitors of *Brassica napus* resistance, as the components of the cell wall of *Plenodomus lingam* (*Leptosphaeria maculans*) have been shown to increase the resistance of this plant [90].

Therefore, further studies will be aimed at gathering knowledge on the exact composition of exopolymers produced by *P. lingam* and *P. biglobosus*, especially sugar monomers in the polysaccharide part, and the type of bonds connecting them. It is highly interesting whether *Plenodomus* exopolymers aggravate the disease symptoms or, on the contrary, induce the resistance of oilseed rape and protect this plant against infection by other pathogens, e.g., by the “sibling” species. It is also important to recognise whether EPSs trigger any signalling pathways in the oilseed rape plant. If so, is this the salicylic acid-dependent pathway active mainly against biotrophic pathogens, or rather the jasmonic acid signalling pathway effective against necrotrophic phytopathogens [29]? *Plenodomus* fungi have a hemi-biotrophic phase, and then they turn to necrotrophy, so both options are possible [31]. A thorough study of the course of immunity induction and pathogenesis-related (PR) protein activity as markers of the SA pathway will clarify whether the protection of oilseed rape against *Plenodomus* employs the SA and JA pathways. It is also unknown whether they act antagonistically or interact with each other in the production of defence substances, e.g., flavonoid phytoalexins, which was detected in the case of rust infection in woody perennial *Populus* [91,92]. The function and the diversity of EPS in *Plenodomus* is still a mystery waiting to be uncovered.

## 5. Conclusions

*Plenodomus lingam* and *P. biglobosus* species are harmful pathogens of cabbage and oilseed rape, causing large economic losses. A thorough understanding of the secondary metabolism and significant differences between these species may ensure better control of these phytopathogens. In this study, we have clearly demonstrated that both species synthesise a number of secondary metabolites, including Extracellular Polymeric Substances. Both species differ considerably in their ability to produce secondary metabolites. This work is the first step, and a further study is needed to provide a clearer insight into *Plenodomus*–oilseed rape interactions.

## Figures and Tables

**Table 1:** Characterisation of winter oilseed rape (*Brassica napus* L.) varieties used in this study.

No.	Cultivar Name	Type of Variety and Resistance	Breeder	Year of Introduction to NLI APV ^1^
1	Birdy	open pollinated	KWS Momont Recherche SARL	2016
2	Bono		HR Smolice, Grupa IHAR	2020
3	Californium		Monsanto Technology LLC	2002/2021 *
4	Gemini		HR Strzelce, Grupa IHAR	2019
5	SY Ilona		Syngenta Participations AG	2016
6	Absolut F1	hybrid, *Rlm7*	Limagrain Europe S.A.S.	2018
7	LG Anarion F1		Limagrain Europe S.A.S.	2020
8	LG Areti F1		Limagrain Europe S.A.S.	2020
9	Luciano KWS F1		KWS Saat SE & Co. KGaA	2019
10	Dominator F1	hybrid, APR37 (*RlmS*)	Deutsche Saatveredelung AG (DSV)	2019
11	Akilah F1		Deutsche Saatveredelung AG (DSV)	2020
12	Kicker F1		Norddeutsche Pflanzenzucht Hans-Georg Lembke KG (NPZ)	2017

^1^ NLI APV—the Polish National List of Agricultural Plant Varieties is an official document containing agricultural plant varieties whose seed/nursery material is eligible for production and marketing in Poland and in European Union territory after their admission to the Common Catalogue; the registry is held by the Research Centre for Cultivar Testing (COBORU), Słupia Wielka, Poland. *—year of deletion from NLI; *Rlm7*—resistance gene; APR37 (*RlmS*)—Adult Plant Resistance derived from *Brassica rapa.*

**Table 2:** Mean *Plenodomus* spp. disease severity in the cultivars of winter oilseed rape. Standard deviations are shown as ± sign (*n* = 3).

		*Plenodomus lingam*		*Plenodomus biglobosus*	
		Highest EPS Yield	Lowest EPS Yield	Highest EPS Yield	Lowest EPS Yield
No.	Cultivar Name	PLIGR3	PLIGR2	PBIGR2	PBIGR3
1	Birdy	3.50 ± 0.51	4.05 ± 0.39	4.00 ± 0.34	2.00 ± 0.56
2	Bono	4.58 ± 0.51	4.58 ± 0.51	2.00 ± 0.65	2.00 ± 0.32
3	Californium	4.00 ± 0.32	4.70 ± 0.80	2.00 ± 0.34	2.00 ± 0.33
4	Gemini	4.00 ± 0.32	3.00 ± 0.32	2.00 ± 0.65	2.00 ± 0.00
5	SY Ilona	4.16 ± 0.38	3.70 ± 0.57	2.00 ± 0.33	2.00 ± 0.47
6	Absolut F1	2.15 ± 0.49	1.30 ± 0.80	3.57 ± 0.51	3.40 ± 0.50
7	LG Anarion F1	1.00 ± 0.46	1.33 ± 0.91	3.68 ± 1.03	3.25 ± 0.44
8	LG Areti F1	1.50 ± 0.51	1.00 ± 0.34	3.68 ± 0.49	2.00 ± 0.84
9	Luciano KWS F1	1.58 ± 0.84	1.30 ± 0.92	3.40 ± 0.60	3.50 ± 0.69
10	Dominator F1	3.00 ± 0.46	2.30 ± 0.57	3.50 ± 0.51	2.50 ± 0.61
11	Akilah F1	3.56 ± 0.51	3.00 ± 0.32	4.20 ± 0.70	5.00 ± 0.46
12	Kicker F1	2.84 ± 0.96	3.60 ± 0.82	4.50 ± 0.76	3.50 ± 0.69
	Total score	**35.89**	**33.86**	**38.50**	**33.15**

**Table 3:** Growth rate ratios (ΔT) of *Plenodomus lingam* and *P. biglobosus* on selected media. Standard deviations are shown as ± sign (*n* = 3).

Fungal Strains	Growth Rate Ratio (ΔT) (mm/Day)				
	A. Siderophores (CAS Agar)	B. Amylolytic (AM Agar)	C. Cellulolytic (CMC Agar)	D. Phosphate Solubilisation (PS Agar)	E. Proteolytic (SM Agar)
PLIGR1	0.035 ± 0.003	0.240 ± 0.01	0.492 ± 0.08	3.377 ± 0.35	6.503 ± 0.35
PLIGR2	0.035 ± 0.004	0.726 ± 0.03	0.407 ± 0.01	1.587 ± 0.11	7.246 ± 0.31
PLIGR3	0.035 ± 0.002	0.240 ± 0.02	0.407 ± 0.02	1.266 ± 0.19	5.458 ± 0.12
PBIGR1	0.467 ± 0.09	0.570 ± 0.02	0.116 ± 0.01	7.305 ± 0.29	12.486 ± 0.4
PBIGR2	0.407 ± 0.03	0.361 ± 0.03	0.407 ± 0.02	5.289 ± 0.22	5.128 ± 0.17
PBIGR3	0.790 ± 0.02	0.361 ± 0.03	0.327 ± 0.02	5.524 ± 0.21	10.140 ± 0.65

**Table 4:** Efficiency of utilisation of individual substrates by fungal strains of *Plenodomus lingam* (PLIGR1, PLIGR2, PLIGR3) and *Plenodomus biglobosus* (PBIGR1, PBIGR2, PBIGR3) on screening media.

Fungal Strains	Efficiency of Activity				
	A. Siderophores (CAS Agar)	B. Amylolytic (AM Agar)	C. Cellulolytic (CMC Agar)	D. Phosphate Solubilisation (PS Agar)	E. Proteolytic (SM Agar)
PLIGR1	+++	++	++	−	−
PLIGR2	+++	+	++	−	−
PLIGR3	+++	++	++	−	−
PBIGR1	+++++	+++	++++	−	−
PBIGR2	+++++	++	+++	−	−
PBIGR3	++++	++	+++	−	−

## Data Availability

Raw data are available on reasonable request due to privacy or ethical restrictions.

## Supplementary Materials

The following supporting information can be downloaded at: https://www.mdpi.com/article/10.3390/metabo13060759/s1. Figure S1. IMASCORE rating scale 0–6 used to evaluate the disease severity symptoms on cotyledons of oilseed rape caused by *Leptosphaeria maculans* (*Plenodomus lingam*). Figure S2. Production of Extracellular Polymeric Substances (g/L) by the isolates of *Plenodomus lingam* (PLIGR2, PLIGR3) and *P. biglobosus* (PBIGR2, PBIGR3): (a) total production from 14 measurements from days 4 to 17; (b) median value; (c) mean production; (d) the highest production in the tested conditions. Figure S3. Severity of phoma leaf spotting caused by the isolates of *Plenodomus lingam* (PLIGR2, PLIGR3) and *P. biglobosus* (PBIGR2, PBIGR3) in the cotyledon test carried out under controlled environment conditions and evaluated at 14 dpi using 0–6 scale: (a) total disease severity score; (b) median value; (c) mean disease severity; (d) the highest mean disease severity score in the tested conditions.
