# Supplementary material for: Differences in the Production of Extracellular Polymeric Substances (EPS) and Other Metabolites of Plenodomus (Leptosphaeria) Infecting Winter Oilseed Rape (Brassica napus L.)
Source: Metabolites. 2023 Jun 17;13(6):759. doi: 10.3390/metabo13060759 (PMC10302272; doi:10.3390/metabo13060759)
Supplement: Supplementary file 1 [file metabolites-13-00759-s001.zip › metabolites-2400639-supplementary.pdf]

metabolites

MDPI

Article

Differences in the Production of Extracellular Polymeric Substances (EPS) and Other Metabolites of *Plenodomus* (*Leptosphaeria*) Infecting Winter Oilseed Rape (*Brassica napus* L.)

Artur Nowak [1], Mateusz Kutyła [1], Joanna Kaczmarek [2], Jolanta Jaroszuk-Ściseł [1,*] and Małgorzata Jędryczka [2,*]

[1] Department of Industrial and Environmental Microbiology, Institute of Biological Sciences, Faculty of Biology and Biotechnology, Maria Curie-Skłodowska University, Akademicka 19, 20-033 Lublin, Poland
[2] Department of Pathogen Genetics and Plant Resistance, Institute of Plant Genetics, Polish Academy of Sciences, Strzeszyńska 34, 60-479 Poznań, Poland
* Correspondence: jolanta.jaroszuk-scisel@mail.umcs.pl (J.J.-Ś.); mjed@igr.poznan.pl (M.J.)

Supplementary Materials

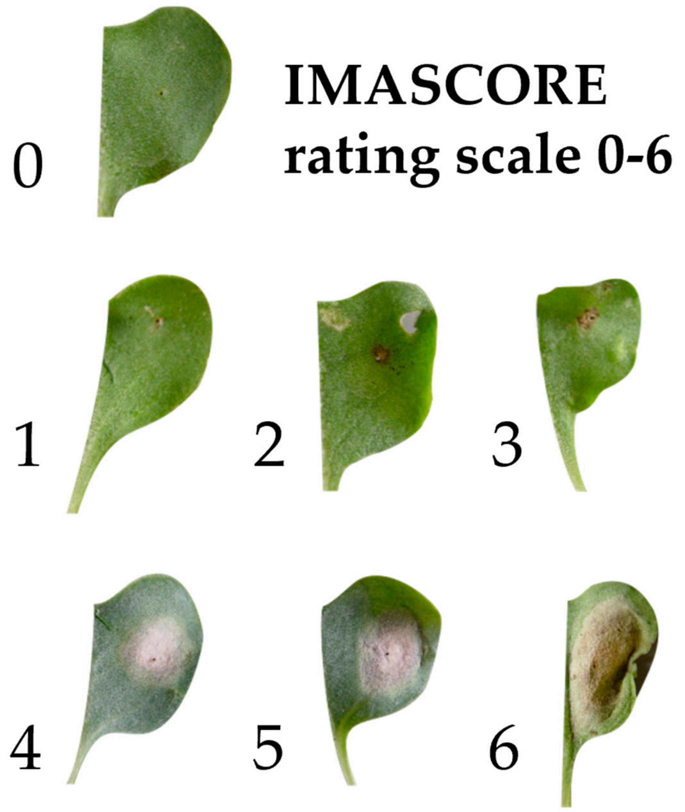

Figure S1. IMASCORE rating scale 0-6 used to evaluate the disease severity symptoms on cotyledons of oilseed rape caused by *Leptosphaeria maculans* (*Plenodomus lingam*).

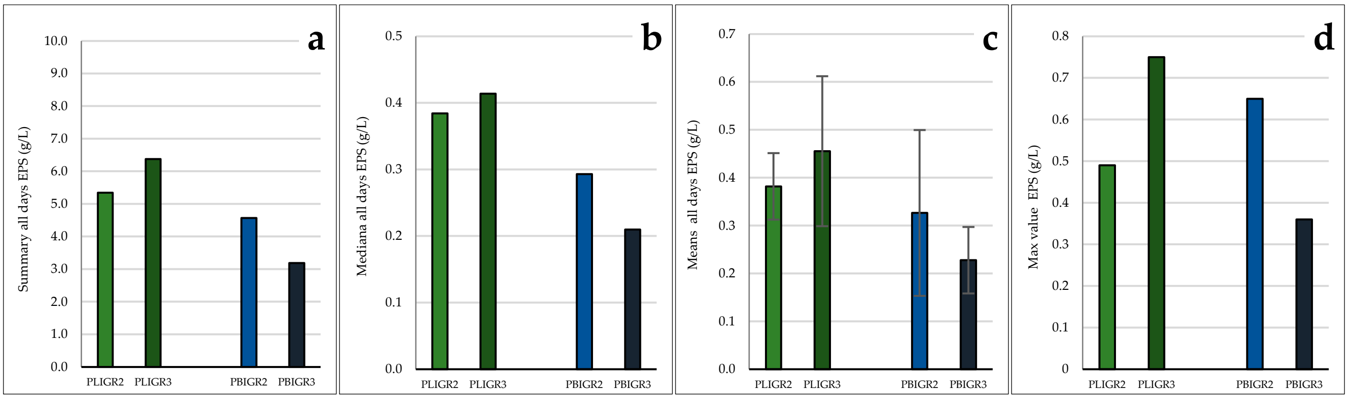

Figure S2. Production of Extracellular Polymeric Substances (g/L) by the isolates of *Plenodomus lingam* (PLIGR2, PLIGR3) and *P. biglobosus* (PBIGR2, PBIGR3): a) total production of 14 measurements from days 4 to 17; b) median value; c) mean production; d) the highest production in the tested conditions.

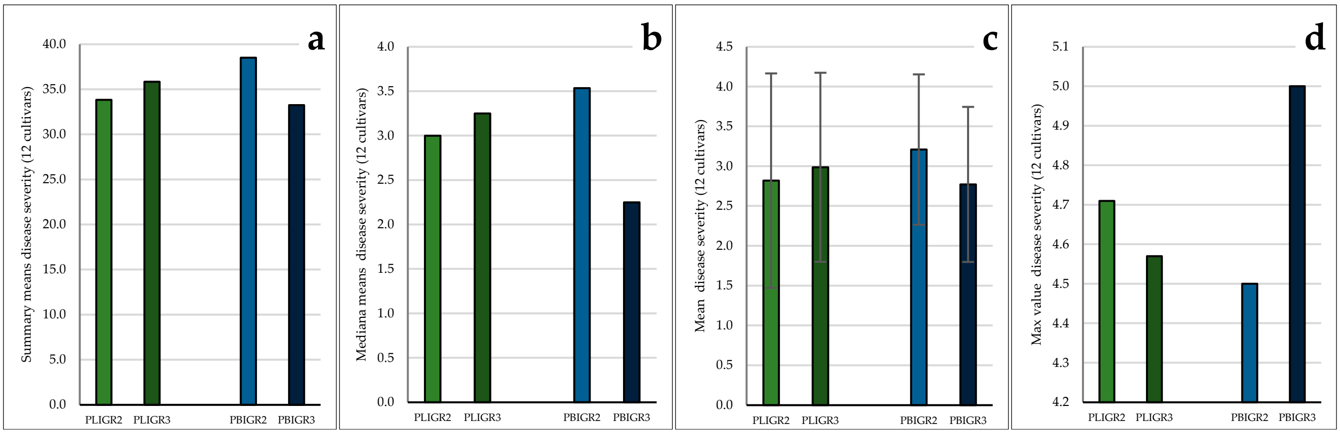

Figure S3. Severity of phoma leaf spotting caused by the isolates of *Plenodomus lingam* (PLIGR2, PLIGR3) and *P. biglobosus* (PBIGR2, PBIGR3) in the cotyledon test carried out in controlled environment conditions and evaluated at 14 dpi using 0-6 scale: a) total disease severity score; b) median value; c) mean disease severity; d) the highest mean disease severity score in the tested conditions.
